# Analyzing the Housing Consumer Preferences via Analytic Hierarchy Process (AHP) in Dubai, United Arab Emirates

**DOI:** 10.3390/bs12090327

**Published:** 2022-09-09

**Authors:** Chuloh Jung, Nahla Al Qassimi, Naglaa Sami Abdelaziz Mahmoud, Sang Yeal Lee

**Affiliations:** 1 Department of Architecture, College of Architecture, Art and Design, Healthy & Sustainable Built Environment Research Center, Ajman University, Ajman P.O. Box 346, United Arab Emirates; 2 Department of Interior Design, College of Architecture, Art and Design, Healthy & Sustainable Built Environment Research Center, Ajman University, Ajman P.O. Box 346, United Arab Emirates; 3 College of Mass Communication, Ajman University, Ajman P.O. Box 346, United Arab Emirates

**Keywords:** housing determinants, consumer preferences, analytic hierarchy process (AHP), Dubai, investment value (IV)

## Abstract

Dubai was one of the top three real estate destinations in the world for investment in 2020. This paper aims to understand the order of preference for various housing determinants by housing consumers in Dubai. As a methodology, a survey was conducted on Dubai residents, and Analytic Hierarchy Process (AHP) was performed to identify the housing determinants and consumers’ preferences. In addition, the respondents’ demographic characteristics identified priorities by income, place of residence, age, gender, and type of house. The results showed that housing consumers place importance on housing price and rent (0.0918), and the investment value (0.0866). However, there was no serious consideration for social and psychological factors, other than safety (0.0730). Regarding gender, men place more importance on the housing price and rent (0.113), and the investment value (0.110). In comparison, women place more importance on factors such as the convenience of transportation (0.104), safety (0.093), and residential environment (0.082). In the age groups, the interest in the educational environment (0.081) among the 40-year-olds was relatively high. In terms of monthly income, the higher the income, the higher the interest in investment value (0.086).

## 1. Introduction

Dubai in the United Arab Emirates (UAE) has a fancy reputation as a continuously growing city, from a small desert port to a renowned metropolitan skyscraper city with a flamboyant lifestyle [1,2]. The rapid development of Dubai was researched in papers many times with a focus on prominent consumption. However, no research was found on why consumers chose to buy or rent housing in Dubai [3,4]. In the last two decades, Dubai has propagated the image of a metropolitan city with constant sprawling developments of mega projects, and Dubai’s high-rise residential buildings have become attractive to housing consumers [5]. In tandem with its luxury image, Dubai real estate offers a better return on investment (ROI) on properties than other major international cities [6].

Dubai is one of the top three real estate destinations in the world for investment in 2020 despite the pandemic [7,8]. According to the 2020 annual transaction report from the Dubai Land Department (DLD), Dubai’s real estate completed 51,414 transactions in 2020, representing a value of approximately 175 billion AED, where 35.6 billion AED was derived from 24,666 foreign investments from Indian, Chinese, British, Pakistani, and French investors [9]. International housing consumers include 6704 from the Gulf Cooperation Council (GCC), such as the Emirati, Saudi, Kuwaiti, Omani, and Bahraini buyers who invested 14.8 billion AED for 8659 residences in Dubai. In 2020, another 4388 Arab nationalities as housing consumers spent 7.5 billion AED for 5283 residences [10].

Based on common sense knowledge, a few obvious factors make Dubai’s property market attractive for housing consumers. First, many studies by real-estate consulting firms show that the average yearly return on Dubai housing is 5.19%, which is significantly higher than other global metropolitan cities [11]. Second, according to the Global Property Guide, the housing price per sqm in Dubai (5918 USD) is far lower compared to other international cities, such as Hong Kong (28,570 USD), London (26,262 USD), and New York (17,191 USD) [12]. Third, property tax in Dubai is 0%, compared to UK (2.53%) and France (1.70%) [13]. Fourth, according to Gallup Law and Order Index 2020, UAE is the fourth safest country in the world compared to Germany (26th), France (31st), the US (36th), and the UK (49th) [14]. Fifth, according to Property Finder, for properties valued above 1 million AED (272,200 USD), the housing consumer could obtain a 2-year residency visa. For above 5 million AED (1.3 million USD), the housing consumer could obtain a 5-year residency visa [15].

However, housing consumers consider various factors when choosing housing as a living environment. Therefore, housing choice behavior can be said to be the result of considering multiple housing determinants in a complex way [16]. As in the general decision-making process, when deciding on housing, it can be seen that housing consumers make a final decision by giving more importance to specific factors (through the process of assigning weights) rather than considering all factors equally [17,18,19].

This study aims to identify the preferences of housing determinants considered by housing consumers in Dubai. More specifically, this study seeks to understand the order of importance for various housing determinants considered by housing consumers and the differences in preference according to consumer characteristics in Dubai.

Due to the recent improvement in living standards and changes in social trends, factors not previously considered necessary are being newly regarded as determinants of housing [20]. Therefore, identifying housing determinants is essential from the perspective of housing developers in Dubai, such as EMAAR, DAMAC, or NAKHEEL, in that they understand market trends by understanding the preferences of housing consumers. From the point of view of Dubai Municipality, it has an essential meaning in terms of providing primary data for changing the policy direction from the existing quantitative supply policy to the qualitative aspect [21].

This study conducted a survey on Dubai residents who were judged to reflect the housing consumers’ recent preferences best. Analytic Hierarchy Process (AHP) was completed to identify the housing determinants and consumers’ preferences for the housing determinants [22,23]. In addition, by using the demographic characteristics of the respondents identified in the survey, preference characteristics by income, place of residence, age, gender, and type of house in which they currently reside were identified [24].

## 2. Materials and Methods

Most of the previous, related studies surveyed apartment residents [25,26,27,28] and focused on satisfaction surveys [29,30,31,32]. The study focusing on apartments is meaningful because the proportion of apartments in the total housing type is high. However, it contradicts the recent trend to diversify housing types. In addition, it can be seen that the meaning of a satisfaction survey on the current residence is not a direct survey on preferred housing. It can also be seen that an in-depth study on the external environment is needed since many existing studies focus on the characteristics inside the house rather than the neighborhood characteristics [33,34]. Considering the limitations of these current findings, this study investigated the preference for neighborhood characteristics among the housing determinants as a general theory for all housing types (Table 1).

In most of the previous studies, regression analysis was performed by setting housing satisfaction as the dependent variable and the housing determinant as an independent variable [35,36]. Since housing satisfaction was not considered in this study, AHP was used instead of regression analysis. It is considered advantageous to identify consumers’ preferences by group and in sequence when AHP is used to determine housing determinants [37].

In this study, the housing determinants were derived by examining the contents of previous studies (Table 2). After reviewing all of the relevant factors, the housing determinants were confirmed in line with the direction of this study, focusing on the neighborhood environment.

Many of the previous studies used AHP [38,39,40]. The differences between the existing studies using AHP and this study are as follows. First, in the case of Choi (2013), the survey was conducted using experts, but this study surveyed housing consumers [41]. All of the three other studies conducted surveys on housing consumers. Cho et al. (2012) have limitations in that they conducted surveys using university students who cannot be called potential housing consumers [42]. The studies of Obeidat et al. (2018) and Lepkova et al. (2016) were conducted in smaller size cities (Amman and Vilnius), so it is judged that there are certain limitations in understanding the overall tendency of housing consumers in metropolitan urban contexts [43,44]. In this study, the results were derived by conducting a survey and analysis targeting the residents in their 30s and older in Dubai, which best reflect the housing consumers’ recent preferences [45].

This study surveyed Dubai residents in their 30s and older who were likely to be interested in purchasing a house [46]. The survey was conducted from 1 September to 31 November 2020, in the vicinity of Dubai Mall (Dubai Downtown), Marina Mall (Dubai Marina), and the Mall of the Emirates (Umm Suqeim). It was decided to conduct a survey that best reflects the population, given that these areas are the most crowded places in Dubai [47]. The total number of questionnaires finally collected was 400 copies, and 342 copies were judged to be valid questionnaires through a consistency test for individual questionnaires, and an analysis was conducted on them.

### Research Concepts Framework

Analytic Hierarchy Process (AHP) is a decision-making method that systematically evaluates mutually exclusive alternatives and derives priorities [48,49]. It is based on the principle that when a problem is complex and there are multiple evaluation criteria, the human brain makes a judgment by using the process of analyzing step by step or hierarchically [50]. It is a method of deriving quantitative results by measuring relative importance or preference on a ratio scale. It can be applied to various practical problems in recent urban design, urban planning, and urban policy fields [51,52]. AHP analysis is a method of stratifying complex problems, decomposing them into major factors and sub-factors, and calculating relative importance based on data generated through a one-to-one comparison of these factors [53]. AHP is a valuable decision-making method when many evaluation criteria or the importance of a component must be prioritized. It can be applied to topics that are difficult to quantify [54,55]. AHP is a method of solving complex decision-making problems through expert judgment and mathematical analysis [56]. It has the advantage of alleviating practical issues, such as time and cost, for reaching consensus without being influenced by the influence of a specific person in group decision-making in which experts participate [57].

**Figure 1:**
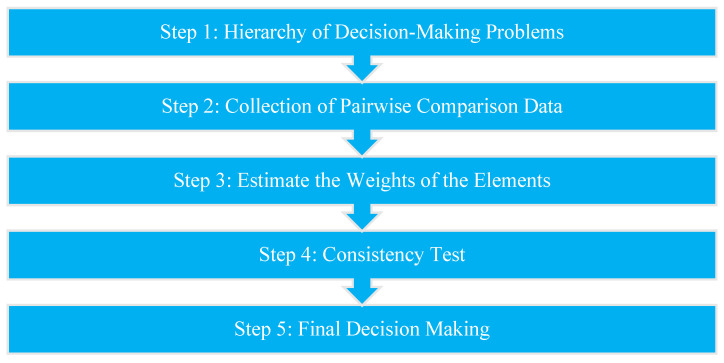
Decision-Making Process via AHP.

Benítez et al. (2012) described the decision-making process through AHP in five steps: identifying the hierarchy of decision-making problems, then collecting pairwise comparison data, followed by estimating the weights of the elements, then, ensuring the consistency of the test, and finally, making the decision, as shown in Figure 1 [58].

**Figure 2:**
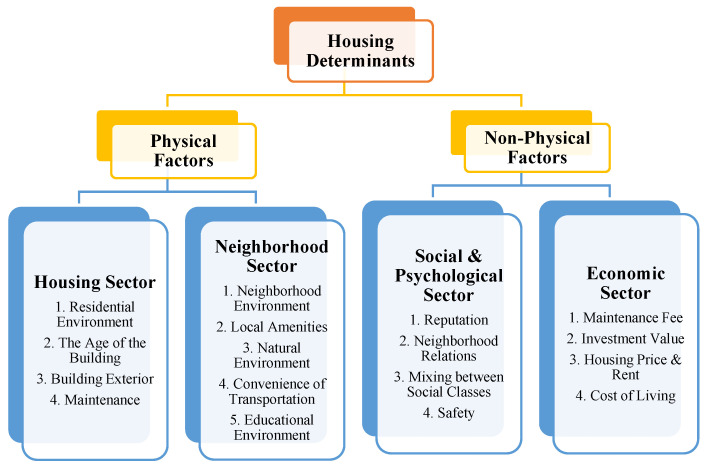
Analysis Model of Research.

Based on this, in this study, the housing determinants were derived through the review of previous studies, and they were hierarchically structured in four stages (Figure 2) [59]. Table 3 and the questionnaire provide definitions of the residential determinants used in the pairwise comparisons. The comparison items were written in conceptual terms, and specific details were added at the bottom of the questionnaire. For example, in this study, ‘residential environment’ means orientation, view, ventilation, and comfort. This study adopted a relatively comprehensive and simple hierarchical structure without dividing the housing determinants in detail to obtain an easy response since the survey was conducted for the general public [60,61].

As mentioned, the survey was conducted among 400 Dubai residents over 30-years-old. The questionnaire was prepared in a pairwise comparison method based on a 5-point Likert scale for each item. The weight of each element was estimated for each layer, and a consistency test was performed [62]. As a result of the reliability test, the consistency ratio was less than 0.1 in all of the categories, confirming that the responses were constant (Table 4) [63]. The weights and rankings obtained from the survey will be presented to all of the respondents as study groups.

## 3. Results

### 3.1. The Characteristics of Survey Participants

Table 5 summarizes the characteristics of the 342 survey respondents who passed the consistency test. The respondent characteristics are then used as data for comparison between groups. As a result of the comparative analysis, there were no remarkable differences between the Dubai Mall (Dubai Downtown), Marina Mall (Dubai Marina), and the Mall of the Emirates (Umm Suqeim) regions. Therefore, this study’s comparative analysis was performed on only the remaining characteristics.

It is worth mentioning that, from Table 5, the number of participants joining the survey from males was almost double that of the females, while the age of the participants was equally distributed among those in their 30s, 40s, and 50s. The number of participants living in apartments was double that of those living in villas. Among the participants, 57.9% owned their housing vs. 42.1% who rented them with an average monthly income of 15.000–20.000 AED.

### 3.2. Determining the Priorities and Weights of Each Sector of Housing Determinants

Table 6 shows the ranking among the items by analyzing the questionnaire on the housing determinants using AHP and determining the weights. The top elements consist of physical and non-physical factors leading to the middle elements. The physical factors are divided into the housing and the neighborhood sectors, while the non-physical factors are divided into social and psychological and economic sectors. The physical factors consist of nine determinants as the bottom elements, and the non-physical factors consist of eight determinants as the bottom elements.

#### 3.2.1. Top Elements Sector

In the top elements, it can be seen that the physical factors (0.543) are considered more critical than the non-physical factors (0.457).

#### 3.2.2. Middle Elements Sector

In middle elements, the neighborhood sector (0.582) was analyzed to be more important than the housing sector (0.418), and the economic sector (0.652) was more important than the social and psychological sector (0.348). It can be seen that the degree of interest in the social and psychological sector is declining.

#### 3.2.3. Bottom Elements Sector

In bottom elements, it can be seen that each sector has a high interest in items such as residential environment (0.373), the convenience of transportation (0.279), safety (0.508), housing price and rent (0.343), and the investment value (0.323). In general, it can be seen that detailed factors corresponding to the economic sector (0.652) (housing price and rent (0.343) and investment value (0.323)) are considered very important even though the principal elements have a small weight.

On the other hand, it can be seen that the consideration of the social and psychological sectors (0.348) excluding safety (0.508) is relatively low. As a result, it is understood and shown that physical factors that can be confirmed and felt are considered more important than psychological factors that are abstract and somewhat ambiguous in determining a residence. These survey results show that housing consumers have a low interest in the community when choosing a place to live [64].

### 3.3. Comparative AHP Analysis of Gender Preference

The respondents were divided by gender, and their preferences are compared in Table 7. Men were more interested in the economic factors of housing, such as housing cost (housing price, rent) and investment value [65]. However, it was found that women are deeply interested in various factors such as the convenience of movement, safety, housing cost, residential environment, and investment value. These results show that, generally, men spend less time at home than women, so they are more interested in the economic aspects of housing, such as housing cost and investment value, than in the residential environment.

On the other hand, it can be seen that women consider mobility and safety as essential factors. As a result, when deciding where to live, it can be inferred that women give more importance to the distance from work to avoid long-distance driving, the convenience of using public transportation to use home services such as maids, and the convenience of using neighborhood facilities for shopping. The importance of women’s safety is reflected in the survey results, indicating that women are more vulnerable to crime than men and consider safety to be relatively important. Although women are also highly interested in economic aspects, it can be seen that their priorities are relatively lower than men.

### 3.4. Comparative AHP Analysis of Age Group Preference

Looking at the factors that determine housing by age group (Table 8), the interest in investment value increases with age. The reason the interest in investment value is higher in the older age group than in the lower age group is presumed to be that the respondents in the older age group have relatively more economic power [66]. The comparison of average household income by age determines that the older a person is, the higher their income is—10,788 AED in the 30s, 18,743 AED in the 40s, and over 23,054 AED in their 50s [67,68]. The ANOVA test confirmed that the income differed between those in their 30s and 50s at the 99% confidence level [69].

Therefore, it is judged that in the case of people in their 30s with low income, interest in housing costs has to be high in the first place, given that housing is a relatively expensive product. However, in the case of people in their 50s with relatively high economic power, the interest in the investment value is relatively high.

People in their 30s are more interested in the convenience of transportation (0.101) than other age groups (0.085 and 0.065). Such interest is also the result of differences in economic power between generations. The lower the economic power, the higher the dependence on public transportation, so it is judged that the convenience of transport was considered significant. It might be said that the younger the group, the more active they are, so the convenience of transportation is essential, but there is no clear evidence for that.

Meanwhile, for people in their 40s, their interest in the educational environment was ranked as 4 (0.081), which is higher than the other two groups, where it was ranked as 7 (0.067) and ranked as 8 (0.059), respectively. This result evidencing a high level of interest in education is obviously because the children of respondents in their 40s are likely to be teenagers.

### 3.5. Comparative AHP Analysis of Monthly Income Preference

The income per household was divided into three groups: 10,000–15,000 AED, 15,000–20,000 AED, and above 20,000 AED (Table 9). As a result of the survey, it was found that all three groups considered investment value (0.086), the convenience of movement (0.085), and housing price and rent (0.093) as important. As a characteristic feature, it was confirmed that the interest in investment value was slightly higher in the group with above 20,000 AED (0.126) compared to 10,000–15,000 AED group (0.068) and 15,000–20,000 AED group (0.102). These data are consistent with the above analysis results, and it can be seen that the higher the income level, the stronger the tendency to think of housing as an investment purpose [70].

### 3.6. Comparative AHP Analysis of Ownership Preference

Homeowners valued investment value (0.098), residential environment (0.095), the convenience of transportation (0.087), house price and rent (0.083) in order of importance. However, tenants considered the significance in the order of housing price and rent (0.139), investment value (0.095), safety (0.094), and educational environment (0.085). The difference between the two groups is found in the housing cost sector (Table 10).

Even those who live in their own houses regard this sector as necessary, but it is judged that the high weight of 0.139 (rank 1) was given because tenants have a much greater sense of the impact of rent. In addition, it was found that the people living in their own houses had a higher interest in the residential environment at 0.095 (rank 2) than the tenants’ 0.069 (rank 6).

This analysis shows that tenants’ awareness of housing is lower than that of self-owners. For those who live in their own house, the weight deviation was narrow from 0.098 (rank 1) to 0.024 (rank 17). On the other hand, the tenants showed a significant deviation from 0.139 (rank 1) to 0.021 (rank 17). The result is that people who own their property are highly interested in their place of residence and give importance to various items. However, it can be seen that the tenants with low awareness of housing are only interested in a few essential factors centered on economic factors.

### 3.7. Comparative AHP Analysis of Housing Type Preference

The housing determinants were analyzed by dividing them into apartment (or townhouse) residents and villa residents (Table 11). There was no significant difference between the two groups. However, it is noteworthy that the villa residents consider the residential environment as the most important (0.108), whereas the apartment (or townhouse) residents have a relatively low awareness of its importance (0.073). It is judged that most of the apartments (or townhouses) have a relatively good living environment compared to villa. On the other hand, it is judged that the results were because the villa residents felt a greater desire to improve the residential environment. It is believed that the relatively high interest in the education environment of apartment residents (0.082) reflects the increased interest in education of the middle class.

## 4. Discussion

**Figure 3:**
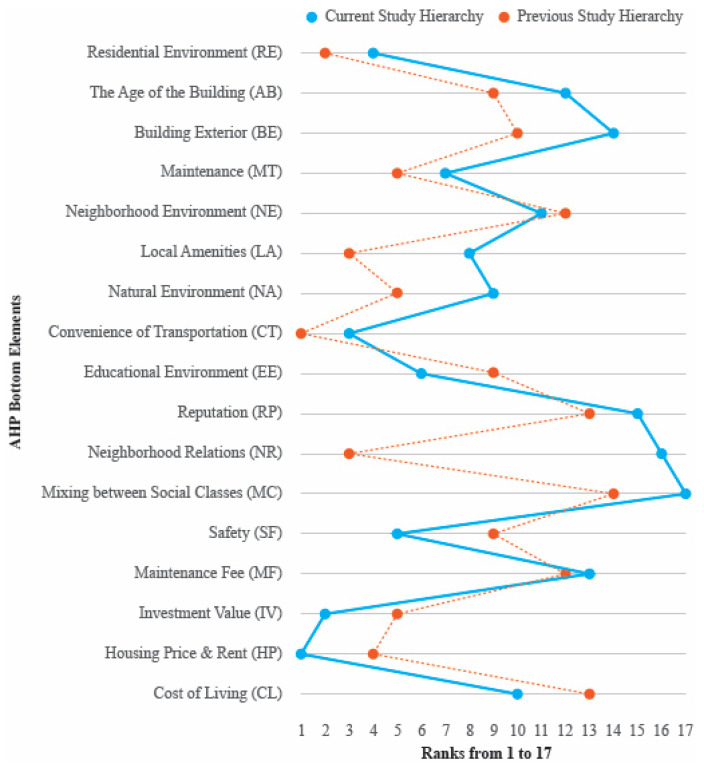
The Comparative Analysis of Hierarchy.

As mentioned above, in this study, the ranking of the factors from 1st to 17th was derived using AHP to identify the housing determinants of Dubai residents. The results were compared with previous studies and the critical factors obtained from the conclusions of previous studies are summarized in Figure 3. Many previous studies did not present the ranking as clearly as in this study [71]. In addition, due to the different relationship between the set housing determinants, a one-to-one comparison was not possible, and the approximate importance was summarized based on the number of essential mentions [72].

First, the analysis results of the previous study and this study are similar to the following. The convenience of transportation (CT) and residential environment (RE) ranked high in both the previous study and the ranking of this study, so it was identified that they were considered necessary to Dubai residents. On the other hand, the reputation/brand of the housing (RP) or mixing between social classes (MC) recorded low rankings in both this study and previous studies, indicating relatively little interest from Dubai residents [73].

Second, compared to other countries, such as in Eastern Europe, the younger generation become less interested in buying housing because of increased housing prices. In Dubai, the 30s only comprise 31 % among the owners of apartments [74].

Third, it is essential to reveal that the housing costs increase with energy costs. In some countries, especially in Europe and currently in UAE, this share of the housing costs makes housing unaffordable, even for households in the possession of housing. In Dubai, the monthly income should be an average of 20.000 AED [75].

The following are the differences between the previous and current studies’ results. In this study, economic factors such as housing price and rent (HP) and investment value (IV) were considered significant. On the other hand, in previous studies, it was analyzed that various factors such as the convenience of transportation (CT), residential environment (RE), local amenities (LA), and relationships between neighbors (NR) were emphasized. The results of this study can be understood as reflecting the current economic situation in Dubai [76].

## 5. Conclusions

Housing is a fundamental right of every Emirati citizen in the United Arab Emirates (UAE), a federation of seven Emirates. Adequate and affordable housing is an essential human requirement, and UAE is approaching housing for its residents from the provider’s perspective. The government exclusively sponsors few initiatives, with little involvement from the commercial, nonprofit, or informal sectors, and the question of their long-term viability looms large. Every stakeholder, including the federal government and the private sector, must approach housing programs to be financially and ecologically sustainable.

A survey was conducted on Dubai residents to determine the factors on which housing consumers place importance when choosing a house, and an AHP analysis was conducted based on the survey results.

In general, it was found that the housing consumers place importance on economic factors, such as investment value (IV) and housing price and rent (HP), when deciding where to live. On the other hand, it was found that there was no serious consideration for the social and psychological factors, other than safety. These results show the characteristics of housing consumers who emphasize specific and visible aspects of housing purchases. Among other factors, the convenience of transportation (CT), residential environment (RE), and educational environment (EE) were identified as important considerations.

As a result of surveying the preferences based on the respondent’s characteristics, it was found that there was no significant difference between the groups. However, gender, age, monthly income, and ownership type found partial differences in the preferences. In terms of gender, the men placed more importance on economic factors, and in comparison, the women placed more importance on factors such as the convenience of transportation (CT), safety (SF), and residential environment (RE). It is understood that these findings reflect the characteristics of this particular group of Dubai residents.

In the case of those aged in their 40s, their interest in the educational environment (EE) was shown to be relatively high, which is seen to be a result of having teenage children. In terms of characteristics by income, it was found that the higher the income, the higher the interest in the investment value (IV).

In the comparison by ownership type, it was found that the self-owners were interested in various items. However, the tenants were focusing their attention on specific factors centered on economic factors. These results can be seen as reflecting the difference in awareness between the owners and the tenants.

The analysis confirmed that the interest of residential consumers was still concentrated on the necessary aspects rather than the qualitative aspects of housing. These results are very similar to the analysis results of previous studies. However, this study confirmed that the Dubai residents’ interest in the economic aspects was higher than the results of previous studies, and the interest in the relationship between neighbors was significantly lowered. It can be understood that this reflects the economic situation of Dubai.

The results of this study suggest that the developers in Dubai and the Dubai Municipality should strive to improve safety and public transport systems. In addition, it can be seen that the lack of interest in the community aspect is expressed as a weak community consciousness and resident participation, so it can be seen that improvement measures are needed.

A limitation of this study is that the proportion of male respondents is high, and there were few respondents over the age of 60, so there is a possibility that the response results may be slightly biased.

Future studies can investigate other emirates with a broader scope for UAE and therefore participate in updating a housing policy for UAE, as there is now a housing program only. A previous study focused on Ras Al Khaimah, where two national housing programs in the Emirate of Ras Al Khaimah offered subsidies for affordable homeownership—the Sheikh Zayed Housing Program and the President’s Initiative—using interviews with municipal and housing officials and a survey of the housing beneficiaries. In this case, a debate on UAE housing could start emerging.

## Figures and Tables

**Table 1:** The Review of Relevant Previous Research.

Researchers	Year	Contents
Obeidat et al.	2018	- Based on a survey of 305 people in 10 apartments in Amman, Jordan, the overall satisfaction function was estimated through AHP, and the relative importance of all factors affecting the overall satisfaction was identified.
Sweis et al.	2013	- Developed an analysis method to derive improvement priorities for each residential environment element through a housing satisfaction survey.
Kyuin and Dongwoo	2011	- Compared the satisfaction level of each new town for various factors constituting housing satisfaction in the new cities in the metropolitan area; - The establishment of a migration plan and the relationship between each variable were summarized.
Zadkarim and Emari	2011	- Analyzed the correlation between characteristics of apartment complex residents and the qualitative satisfaction of a green residential environment, targeting new towns in the metropolitan area and analyzing the residents’ main tendencies.
Lepkova et al.	2016	- A study on the characteristics of the living environment that affected the housing choice of residents in the Lithuanian housing market; - AHP analysis method was used to investigate and analyze the importance of residents’ housing selection factors.
Cho et al.	2011	- Analyzed the decision-making process of college students on how to choose their future housing in a metropolitan area; - The survey subjects were 80 university students, and the AHP analysis method was used.
Choi	2013	- A method for more accurately measuring apartment housing satisfaction through AHP and factor analysis was presented; - After measuring apartment housing satisfaction, the examination was performed regarding what differences exist in housing satisfaction depending on the apartment type.
Son et al.	2015	- Conducted surveys through visits by investigators; - Checked the influence of past experiences, which are individual characteristics of households, on the evaluation of the current physical environment.
Chang et al.	2015	- The effect of each item’s satisfaction on the overall housing satisfaction was studied by comprehensively analyzing spatial characteristics, primary responses, and item satisfaction by dividing the district in Taiwan.
Rahman et al.	2015	- Analysis of baby boomers’ Housing Satisfaction and Housing Decision Factors; - Traced the variables of their housing demand and suggested a housing supply direction for the old age of baby boomers.
Park et al.	2019	- This study proposed case-based reasoning (CBR) model for estimating the time when the first repair will be needed after the completion of construction; - CBR and fuzzy AHP were employed as research methodologies.
Eryürük et al.	2021	- This study concluded that only one stakeholder should not define the criteria and their weights in a project, but all particular stakeholders should be included during the planning and application process with AHP.
Kim and Han	2012	- This study was on the factors and relationship analysis that affect user satisfaction by parking lot type; - A structural equation model was derived from AHP survey data of apartment residents.

**Table 2:** The Review of Previous Research to Derive Housing Determinants.

Elements	Obeidat et al. (2018)	Sweis et al. (2013)	Kyuin and Dongwoo (2011)	Zadkarim and Emari (2011)	Lepkova et al. (2016)	Cho et al. (2011)	Choi (2013)	Son et al. (2015)	Chang et al. (2015)	Rahman et al. (2015)	Park et al. (2019)	Eryürük et al. (2021)	Kim and Han (2012)
Residential Environment	●	●					●		●				●
The Age of the Building							●	●			●		
Building Exterior		●	●				●						●
Maintenance					●		●			●			
Neighborhood Environment						●			●	●		●	
Local Amenities		●	●	●	●		●			●	●		
Natural Environment	●	●	●	●	●	●	●	●		●	●	●	●
Convenience of Transportation	●	●	●	●	●	●			●	●	●	●	
Educational Environment	●	●	●		●	●	●	●		●	●	●	●
Reputation					●	●	●		●		●		
Neighborhood Relations	●		●		●		●		●	●	●		●
Mixing between Social Classes				●				●			●		
Safety		●	●	●	●		●	●		●	●		
Maintenance Fee			●					●	●			●	●
Investment Value		●	●			●			●	●	●	●	●
Housing Price and Rent	●				●								●
Cost of Living			●				●						
Education Level				●		●		●	●				
Annual Income						●			●				●
Type of Family						●		●	●				●
Type of Ownership					●		●		●	●			

**Table 3:** The Middle and Bottom Elements of Housing Determinants for AHP Analysis.

Middle Elements	Bottom Elements	Descriptions
Housing Sector	Residential Environment	1. Orientation 2. View 3. Ventilation 4. Comfort of Residence
	The Age of the Building	1. Year of Construction 2. Building Safety
	Building Exterior	1. Design 2. Housing Type
	Maintenance	1. Defect Repair, Cleaning/Garbage Disposal
Neighborhood Sector	Neighborhood Environment	1. Kindergarten 2. Playground 3. Parking Convenience 4. Landscape
	Local Amenities	1. Hospital 2. Hypermarket 3. Shopping Mall 4. Public Facilities
	Natural Environment	1. Greenery 2. Park 3. Beach 4. Desert
	Convenience of Transportation	1. Distance from Work 2. Use of Public Transportation
	Educational Environment	1. School District 2. Academy 3. Existence of Entertainment Facilities
Social and Psychological Sector	Reputation	1. Developer Brand 2. Distance from Unpleasant Facility
	Neighborhood Relations	1. Affinity with Neighbors 2. Degree in Socializing
	Mixing between Social Classes	1. Income Level of Local Residents 2. Occupation 3. Social Status
	Safety	1. Security 2. Safety from Disasters
Economic Sector	Maintenance Fee	1. Maintenance Fee
	Investment Value	2. Return on Investment (ROI)
	Housing Price and Rent	3. Stability of House Price & Rent
	Cost of Living	4. Regional Prices

**Table 4:** The Consistency Ratio of Middle Elements for AHP Analysis.

Middle Elements	CR (Consistency Ratio)
Housing Sector	0.0027
Neighborhood Sector	0.0013
Social and Psychological Sector	0.0051
Economic Sector	0.0022

**Table 5:** The Characteristics of Survey Participants.

Category	Subcategory	Number (Percentage)
Gender	Male	220 (64.3)
	Female	122 (35.6)
Age Group	The 30s	106 (31.0)
	The 40s	122 (35.7)
	The 50s	114 (33.3)
Housing Type	Apartment (Townhouse)	224 (65.5)
	Villa	118 (34.5)
Ownership	Own	198 (57.9)
	Rent	144 (42.1)
Monthly Income	10,000–15,000 AED	98 (28.7)
	15,000–20,000 AED	155 (45.3)
	Above 20,000 AED	89 (26.0)
Living Area	Downtown/Marina/Umm Suqeim	128 (37.4)
	Other Area	214 (62.6)

**Table 6:** The Weight of Housing Determinants.

Top Elements	Weight (A)	Middle Elements	Weight (B)	Bottom Elements	Weight (C)	Total Weight (A × B × C)	Rank
Physical Factors	0.543	Housing Sector	0.418	Residential Environment (RE)	0.373	0.0849	4
				The Age of the Building (AB)	0.203	0.0466	12
				Building Exterior (BE)	0.152	0.0342	14
				Maintenance (MT)	0.272	0.0625	7
		Neighborhood Sector	0.582	Neighborhood Environment (NE)	0.152	0.0468	11
				Local Amenities (LA)	0.174	0.0530	8
				Natural Environment (NA)	0.166	0.0510	9
				The convenience of Transportation (CT)	0.279	0.0854	3
				Educational Environment (EE)	0.229	0.0698	6
Non-Physical Factors	0.457	Social and Psychological Sector	0.348	Reputation (RP)	0.191	0.0277	15
				Neighborhood Relations (NR)	0.157	0.0225	16
				Mixing between Social Classes (MC)	0.143	0.0206	17
				Safety (SF)	0.508	0.0730	5
		Economic Sector	0.652	Maintenance Fee (MF)	0.156	0.0422	13
				Investment Value (IV)	0.323	0.0866	2
				Housing Price and Rent (HP)	0.343	0.0918	1
				Cost of Living (CL)	0.179	0.0487	10

**Table 7:** The AHP Analysis of Gender Preference.

Rank	Male (64.3%)		Female (35.6%)		Total (100%)	
	Bottom Elements	Weight	Bottom Elements	Weight	Bottom Elements	Weight
1	HP	0.113	CT	0.104	HP	0.093
2	IV	0.110	SF	0.093	IV	0.086
3	RE	0.087	HP	0.087	CT	0.085
4	CT	0.077	RE	0.082	RE	0.084
5	SF	0.076	IV	0.076	SF	0.074
6	EE	0.075	MT	0.065	EE	0.070
7	MT	0.060	EE	0.060	MT	0.063
8	CL	0.059	LA	0.059	LA	0.052
9	NA	0.056	AB	0.057	NA	0.051
10	LA	0.048	NE	0.053	CL	0.049
11	MF	0.047	MF	0.048	NE	0.048
12	NE	0.043	CL	0.046	AB	0.046
13	AB	0.042	NA	0.044	MF	0.042
14	BE	0.032	BE	0.037	BE	0.035
15	RP	0.030	RP	0.035	RP	0.027
16	NR	0.025	MC	0.029	NR	0.022
17	MC	0.022	NR	0.023	MC	0.021

**Table 8:** The AHP Analysis of Age Group Preference.

Rank	30s (31.0%)		40s (35.7%)		50s (33.3%)		Total (100%)	
	Bottom Elements	Weight	Bottom Elements	Weight	Bottom Elements	Weight	Bottom Elements	Weight
1	HP	0.102	RE	0.096	IV	0.124	HP	0.093
2	CT	0.101	IV	0.092	HP	0.112	IV	0.086
3	IV	0.089	HP	0.091	RE	0.089	CT	0.085
4	SF	0.086	EE	0.081	SF	0.076	RE	0.084
5	RE	0.081	SF	0.079	CT	0.073	SF	0.074
6	MT	0.069	MT	0.072	NA	0.068	EE	0.069
7	EE	0.067	CT	0.065	CL	0.065	MT	0.063
8	CL	0.059	NA	0.057	EE	0.059	LA	0.053
9	LA	0.055	AB	0.055	LA	0.057	NA	0.051
10	AB	0.053	NE	0.053	MF	0.049	CL	0.049
11	MF	0.045	MF	0.049	NE	0.044	NE	0.048
12	NE	0.044	LA	0.046	MT	0.039	AB	0.046
13	NA	0.042	RP	0.038	NR	0.033	MF	0.042
14	BE	0.038	CL	0.037	RP	0.030	BE	0.035
15	RP	0.027	BE	0.035	AB	0.028	RP	0.027
16	MC	0.022	NR	0.029	BE	0.027	NR	0.023
17	NR	0.021	MC	0.026	MC	0.024	MC	0.021

**Table 9:** The AHP Analysis of Monthly Income Preference.

Rank	10,000–15,000 AED (28.7%)		15,000–20,000 AED (45.3%)		Above 20,000 AED (26.0%)		Total (100%)	
	Bottom Elements	Weight	Bottom Elements	Weight	Bottom Elements	Weight	Bottom Elements	Weight
1	RE	0.112	HP	0.107	IV	0.126	HP	0.093
2	CT	0.087	IV	0.102	CT	0.101	IV	0.086
3	HP	0.086	CT	0.085	HP	0.100	CT	0.085
4	MT	0.085	RE	0.082	RE	0.083	RE	0.084
5	SF	0.084	SF	0.081	EE	0.080	SF	0.074
6	IV	0.068	EE	0.071	SF	0.075	EE	0.070
7	AB	0.061	MT	0.064	LA	0.066	MT	0.063
8	CL	0.056	CL	0.054	NA	0.060	LA	0.053
9	EE	0.053	LA	0.053	NE	0.045	NA	0.052
10	LA	0.051	NA	0.052	MF	0.041	CL	0.048
11	NA	0.048	NE	0.048	AB	0.040	NE	0.047
12	BE	0.046	MF	0.047	MT	0.039	AB	0.046
13	MF	0.045	AB	0.044	CL	0.038	MF	0.043
14	NE	0.044	BE	0.034	RP	0.034	BE	0.034
15	RP	0.028	RP	0.031	BE	0.028	RP	0.029
16	NR	0.025	NR	0.025	MC	0.024	NR	0.022
17	MC	0.021	MC	0.021	NR	0.019	MC	0.020

**Table 10:** The AHP Analysis of Ownership Preference.

Rank	Own (57.9%)		Rent (42.1%)		Total (100%)	
	Bottom Elements	Weight	Bottom Elements	Weight	Bottom Elements	Weight
1	IV	0.098	HP	0.139	HP	0.093
2	RE	0.095	IV	0.095	IV	0.086
3	CT	0.087	SF	0.094	CT	0.085
4	HP	0.083	EE	0.085	RE	0.084
5	SF	0.075	CT	0.081	SF	0.074
6	MT	0.065	RE	0.069	EE	0.069
7	EE	0.060	MT	0.056	MT	0.063
8	AB	0.058	CL	0.055	LA	0.053
9	CL	0.054	LA	0.054	NA	0.051
10	NA	0.052	NE	0.049	CL	0.049
11	LA	0.051	NA	0.047	NE	0.048
12	MF	0.048	MF	0.045	AB	0.046
13	NE	0.046	AB	0.033	MF	0.042
14	BE	0.045	RP	0.029	BE	0.035
15	RP	0.033	BE	0.025	RP	0.027
16	NR	0.026	NR	0.022	NR	0.022
17	MC	0.024	MC	0.021	MC	0.021

**Table 11:** The AHP Analysis of Housing Type Preference.

Rank	Apartment/Townhouse (65.5%)		Villa (34.5%)		Total (100%)	
	Bottom Elements	Weight	Bottom Elements	Weight	Bottom Elements	Weight
1	HP	0.101	RE	0.108	HP	0.093
2	IV	0.097	HP	0.101	IV	0.086
3	CT	0.084	IV	0.092	CT	0.085
4	EE	0.082	CT	0.085	RE	0.084
5	SF	0.081	SF	0.084	SF	0.074
6	RE	0.073	MT	0.077	EE	0.070
7	LA	0.058	AB	0.058	MT	0.061
8	MT	0.054	CL	0.054	LA	0.053
9	CL	0.053	EE	0.052	NA	0.051
10	NA	0.051	NA	0.048	CL	0.050
11	MF	0.049	LA	0.045	NE	0.047
12	NE	0.048	NE	0.043	AB	0.046
13	AB	0.038	MF	0.042	MF	0.042
14	RP	0.034	BE	0.039	BE	0.035
15	BE	0.032	RP	0.027	RP	0.027
16	NR	0.026	NR	0.025	NR	0.023
17	MC	0.024	MC	0.022	MC	0.020

## Data Availability

New data were created or analyzed in this study. Data will be shared upon request and consideration of the authors.
